# Mechanobiological Strategies to Enhance Stem Cell Functionality for Regenerative Medicine and Tissue Engineering

**DOI:** 10.3389/fcell.2021.747398

**Published:** 2021-12-03

**Authors:** Muhammad Shafiq, Onaza Ali, Seong-Beom Han, Dong-Hwee Kim

**Affiliations:** ^1^ Department of Biotechnology, Faculty of Life Sciences, University of Central Punjab, Lahore, Pakistan; ^2^ School of Chemistry and Chemical Engineering, Tiangong University, Tianjin, China; ^3^ KU-KIST Graduate School of Converging Science and Technology, Korea University, Seoul, South Korea; ^4^ Department of Integrative Energy Engineering, College of Engineering, Korea University, Seoul, South Korea

**Keywords:** biomaterials, stem cell, mechanobiology, cell fate modulation, immunosuppression, cell therapy, tissue engineering, regenerative medicine

## Abstract

Stem cells have been extensively used in regenerative medicine and tissue engineering; however, they often lose their functionality because of the inflammatory microenvironment. This leads to their poor survival, retention, and engraftment at transplantation sites. Considering the rapid loss of transplanted cells due to poor cell-cell and cell-extracellular matrix (ECM) interactions during transplantation, it has been reasoned that stem cells mainly mediate reparative responses via paracrine mechanisms, including the secretion of extracellular vesicles (EVs). Ameliorating poor cell-cell and cell-ECM interactions may obviate the limitations associated with the poor retention and engraftment of transplanted cells and enable them to mediate tissue repair through the sustained and localized presentation of secreted bioactive cues. Biomaterial-mediated strategies may be leveraged to confer stem cells enhanced immunomodulatory properties, as well as better engraftment and retention at the target site. In these approaches, biomaterials have been exploited to spatiotemporally present bioactive cues to stem cell-laden platforms (e.g., aggregates, microtissues, and tissue-engineered constructs). An array of biomaterials, such as nanoparticles, hydrogels, and scaffolds, has been exploited to facilitate stem cells function at the target site. Additionally, biomaterials can be harnessed to suppress the inflammatory microenvironment to induce enhanced tissue repair. In this review, we summarize biomaterial-based platforms that impact stem cell function for better tissue repair that may have broader implications for the treatment of various diseases as well as tissue regeneration.

## Introduction

**FIGURE 1:**
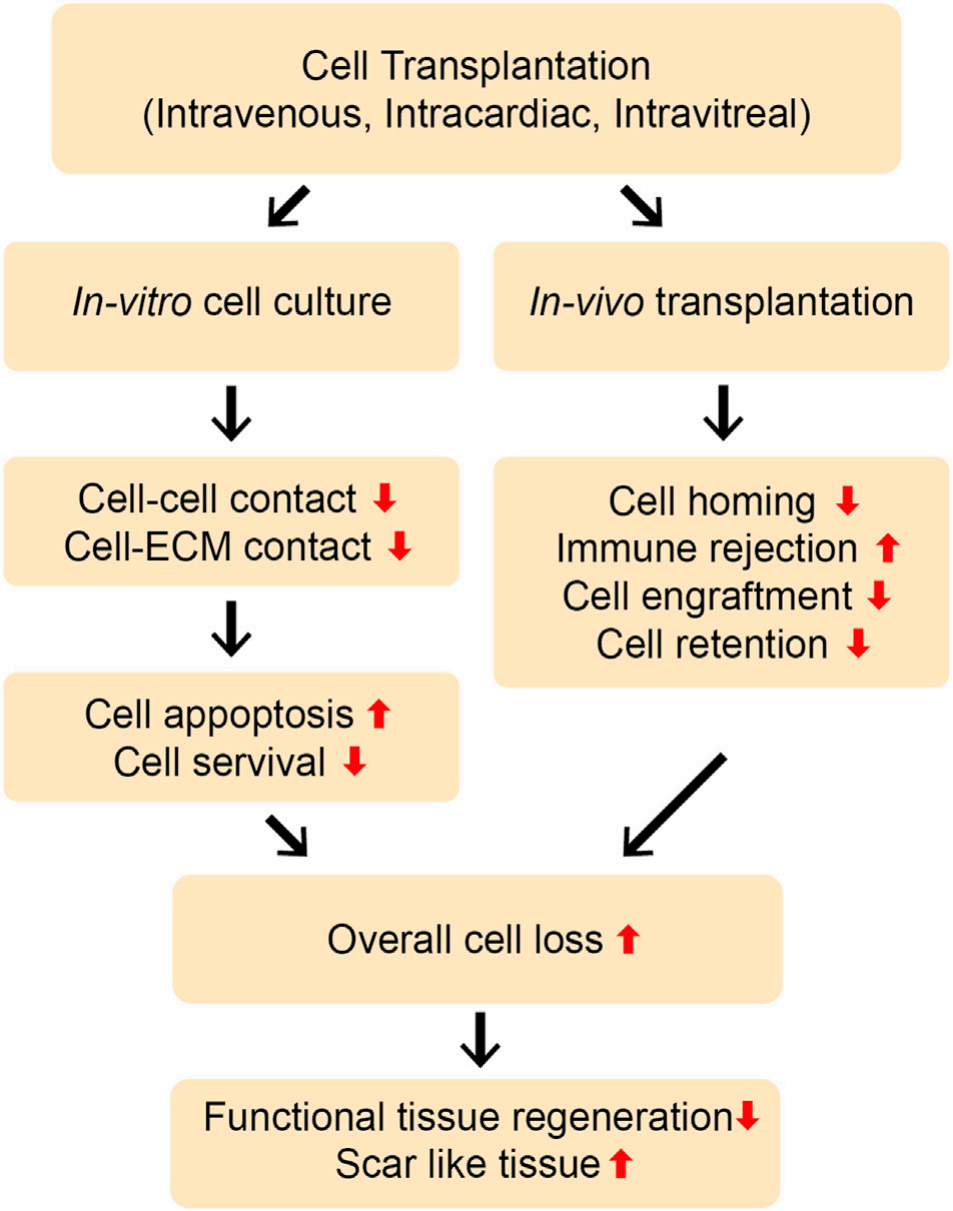
Limitations of direct cell transplantation. Cells can be transplanted *in vivo* by different routes, such as intravenous, intramuscular, intra-cardiac, or intra-vitreous injection. The direct transplantation of stem cells and progenitor cells without using biomaterial platforms may lead to the poor cell-cell and cell-ECM interactions, resulting into the severe loss of the cells through apoptosis *in vitro* and *in vivo*. Moreover, the cells can experience rapid loss upon transplantation *in vivo* due to poor cell survival, retention, and engraftment at the injury site. Poor cell homing and immune rejection may further aggravate this problem. Overall, this can lead to severe cell loss and less functional, more scar-like tissue repair.

Stem cell therapy has garnered extensive attention and been investigated for the treatment of various pathologies. This is mainly because of the multilineage differentiation capability of stem cells, their secretion of paracrine factors, including extracellular vesicles (EVs), exosomes, ectosomes (microparticles/macrovesicles), and their immunomodulatory properties. Consequently, stem cell therapy has been used for the regeneration of a multitude of tissues, such as infarcted myocardium, spinal cord, nerves, and skin, as well as to treat vascular injuries, ocular defects, and melanoma (Karantalis and Hare, 2015; Trounson and McDonald, 2015; Parmar et al., 2020). While implanted stem cells participate in the regenerative process by differentiating into tissue-specific cells, their poor survival and retention *in vivo* hamper tissue repair (Figure 1). Recently, stem cell labeling techniques using traceable nanoparticles and fluorescent dyes have been used to precisely track the survival and integration of transplanted cells in the targeted tissues (Teo et al., 2020). These factors have fueled research to elucidate the involvement of paracrine factors, including EVs in the tissue repair process.

**FIGURE 2:**
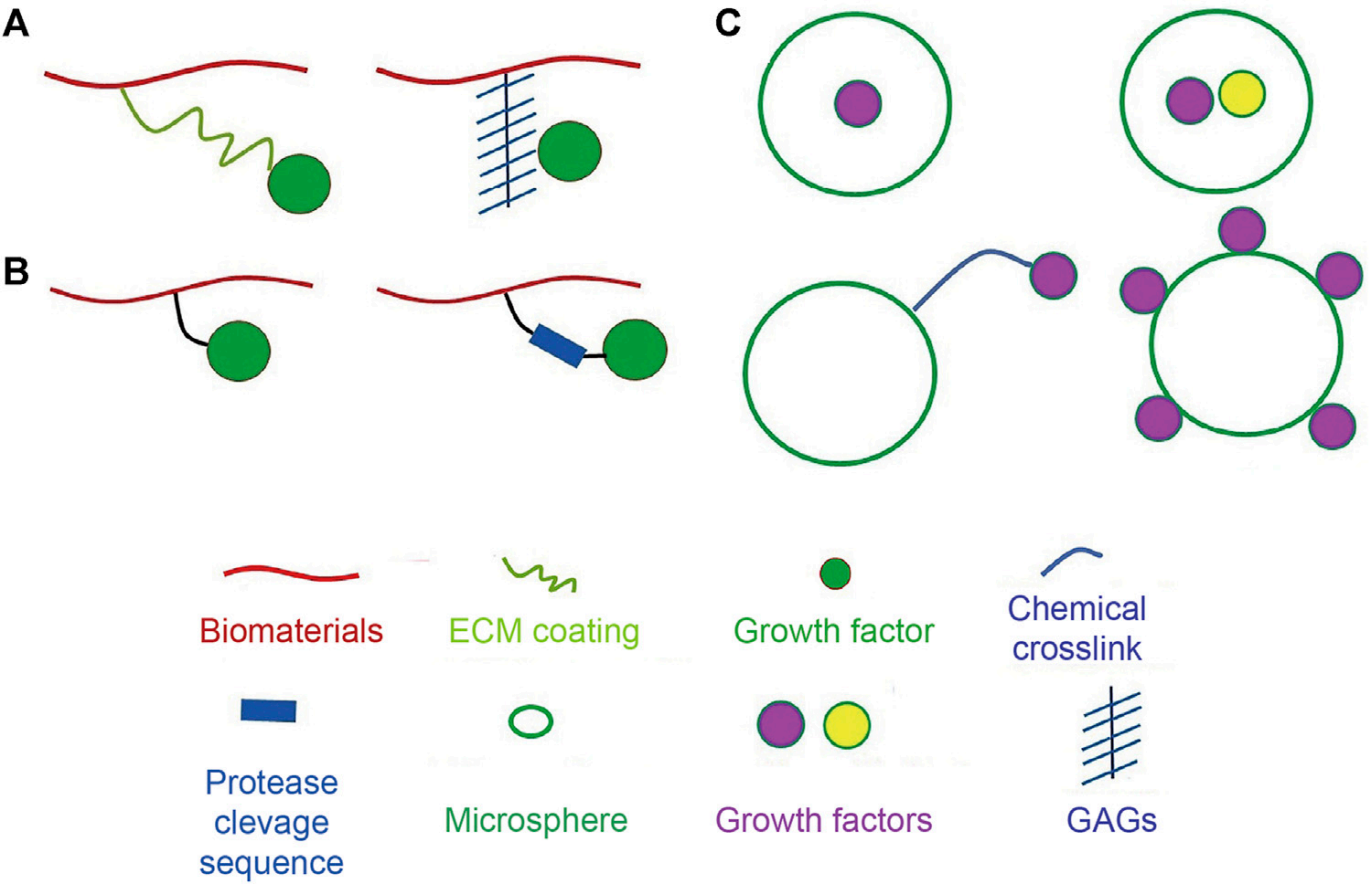
Strategies for attachment of bioactive molecules to biomaterials. **(A)** Strategies to non-covalently attach bioactive molecules to biomaterials using physical tools. Bioactive molecules can be absorbed on the surface of the biomaterial through coating ECM proteins or can be electrostatically associated with biomaterials via heparin linkage. **(B)** Strategies to covalently attach bioactive molecules using chemical tools. Protease-degradable amino acids sequences can be introduced into crosslinks to instruct release of signaling cues. **(C)** Methodologies for incorporation of bioactive molecules to microspheres. Bioactive molecules can be encapsulated, adsorbed on the surface, or chemically linked to the microspheres. Adapted with permission from (Shafiq et al., 2016). Copyrights reserved Elsevier.

Extensive reports have discussed the therapeutic potential of stem cell grafting into injured or defective organs. Improving the survival and retention of transplanted stem cells may help in elucidating the role of cell-mediated paracrine factors, and EVs involved in this process (Table 1) (Jha et al., 2015a; Jha et al., 2015b). Therefore, ensuring stem cell survival during transplantation and *in vivo* is crucial for the success of regenerative medicine and tissue engineering approaches. There is currently a good understanding of cell survival mechanisms, including cell-cell and cell-extracellular matrix (ECM) crosstalk. Studies to address the rapid loss of transplanted cells and their poor survival *in vivo* have used different biomaterial candidates, such as hydrogels, engineered cell sheets, instructive biomaterials, and ECM-mimicking platforms and have improved our understanding of cell loss during and after transplantation (Shafiq et al., 2016). The loss of cell-cell and cell-ECM crosstalk is believed to be the primary cause of cell loss *in vitro*, but cell preconditioning, licensing, and instructive biomaterials could be used to overcome this problem. Additionally, the immunosuppressive microenvironment and inflammatory response at the target site are perceived to compromise cell-mediated functions, and this problem has been addressed by designing bioactive cue-tethered biomaterials (Hettiaratchi et al., 2016), including heparin-conjugated materials to sequester heparin-binding growth factors and interferon-gamma (IFN-γ)-tethered hydrogels for immune suppression (Figure 2). Furthermore, engineering the microenvironment at the site of injury has been pursued to improve the survival and engraftment of transplanted cells. Biomaterial-mediated delivery of cytokines and chemokines, such as transforming growth factor-beta (TGF-β), has been exploited to induce immune suppression in the injury microenvironment (Liu et al., 2016a).

**TABLE 1:** List of abbreviations.

Full name	Abbreviation	Full name	Abbreviation
Glycosaminoglycans	GAGs	Manganese oxide	MnO_2_
Bone morphogenetic protein-2	BMP-2	Brain-derived neurotrophic factor	BDNF
Mesenchymal stem cells	MSCs	Glial-derived neurotrophic factor	GDNF
CXC chemokine receptor 4	CXCR4	Polycaprolactone	PCL
Insulin-like growth factor 1	IGF-1	Neural stem cells	NSCs
Vascular endothelial growth factor	VEGF	Induced-pluripotent stem cells	iPSCs
Interferon-gamma	IFN-γ	Cardiomyocytes	CMs
Hyaluronic acid	HA	Beta cyclodextrin	β-CD
Transforming growth factor-beta	TGF-β	Mitogen-activated protein kinase	MAPK
Monocyte chemoattractant protein 1	MCP-1	Hydrogen peroxide	H_2_O_2_
Interleukin-12	IL-12	Magnesium oxide	MgO_2_
Tumor necrosis factor-alpha	TNF-α	Cardiac stem cells	CSCs
Human umbilical vein endothelial cells	HUVECs	Poly (vinyl alcohol)	PVA
Cardiac progenitor cells	CPCs	Polyethylene glycol	PEG
Smooth muscle cells	SMCs	Polythioketal	PTK
Adipose-derived stem cells	ADSCs	Nanoparticles	NPs
Bioluminescent imaging	BLI	Endothelial progenitor cells	EPCs
Schwann cells	SCs	Extracellular domain-1	ECD-1
Hematopoietic stem cells	HSCs	Poly (N-isopropylacrylamide)	PNIPAM
Poly (L-lactide-co-glycolide)	PLGA	Adhesive protein-based immiscible condensed liquid systems	APICLS
Epigallocatechin gallate	EGCG	Prostaglandin E2	PGE-2

Similarly, the use of instructive biomaterials possessing mechanical properties similar to those of native tissues and incorporating different growth factors has been proposed to enhance the survival and integration of transplanted cells. Because most of the transplanted cells are lost owing to differences in mechanical properties between the hydrogels used for cell transplantation and the loss of cell-cell and cell-ECM contacts, the use of synthetic hydrogels possessing shear-thinning and self-healing characteristics has been pursued to improve the injectability of the cells into tissues *in vivo* in a non-invasive manner. Additionally, to mimic the cell niche-like microenvironment *in vitro*, ECM and ECM-derived peptide sequences and molecules capable of interacting with cellular receptors, such as hyaluronic acid (HA), have been integrated into biomaterials (Somaa et al., 2017). The microenvironment at the injury site leverages signaling cues, such as stromal cell-derived factor 1 alpha (SDF-1α) and CXC chemokine receptor 4 (CXCR4) signaling, to enhance stem cell and progenitor cell mobilization and recruitment. Instructive biomaterials possessing these cues have been shown to improve stem cell function *in vitro* and *in vivo*. However, implementing these methods requires additional strategies such as inducing vascular-like networks into the biomaterials to enhance their cellular integration via anastomosis between the transplanted cells and tissues and the host tissues or organs. These strategies have been extensively detailed elsewhere (Shafiq et al., 2016) and are thus not discussed in this review. The subsequent four sections of this review discuss some of the recent strategies employed to overcome several challenges in stem cell therapy.

## Local Presentation of Bioactive Cues to Enhance Stem Cell Survival and Retention

### Engineered Biomaterials for Protein Sequestration

Biomaterials are engineered materials that interact with host tissues and assist in their smooth functioning or induce the regeneration of injured tissues or organs (Abdulghani and Mitchell, 2019). For instance, dental fillers and other implants, such as those used for the replacement of the hip and joints, to remediate bone and spinal fusion, or for vascular reconstruction, all fall under this category. Depending on their intended application, biomaterials can be either bio-inert, that is, biologically inert to the body or instructive, that is, leveraging biochemical cues for neo-tissue regeneration or the restoration of a lost function (Pavlovic, 2015). Bioinert materials are generally either biocompatible metals, such as titanium, tantalum, or zirconia, or non-degradable polymers, including polymethyl methacrylate (PMMA) and expanded polytetrafluoroethylene (ePTFE). They do not degrade *in vivo*, and may need to be removed at some point after implantation. In contrast, the latter class includes biomaterials that can interact with the host cells and leverage biochemical signals for neo-tissue regeneration or restoration of a lost function. These materials include biocompatible and biodegradable metals such as magnesium, as well as polymeric materials such as poly (L-lactide) (PLLA), polycaprolactone (PCL), polyglycolic acid (PGA), poly (L-lactide-co-glycolic acid) (PLGA), and poly (L-lactide-co-ε-caprolactone) (PLCL). Because tissues and the extracellular matrix (ECM) dynamically interact with the *in vivo* microenvironment, dynamic biomaterials that can reciprocally interact with the *in vivo* microenvironment, while perceiving and leveraging biochemical signals, including sensing force or even remodeling, have recently been proposed (Zhang et al., 2020).

Biomaterial scaffolds should be biocompatible with the host tissues. A multitude of features, including tissue- or organ-specific biophysical properties and bioactive cues, are part of these biomaterials to help them create a conducive environment for tissue regeneration (Zhao et al., 2019). Another crucial aspect is the biodegradability of the biomaterials. The degradation products should not be toxic to avoid inflammatory responses. Biomaterials encompass metals, ceramics, and polymers, and the latter can be either natural or synthetic. Natural polymers, including ECM-like proteins such as fibronectin, laminin, collagen, polysaccharides, and glycosaminoglycans are widely used as biological vehicles and scaffolds because of their biocompatibility and biodegradability. Despite these interesting features, the use of natural polymers is mainly impeded by their poor mechanical properties. An advantage of using synthetic polymers, such as PCL, PLCL, and PLGA is that good control can be exercised over their synthesis and modification (Cameron and Shaver, 2011). Because synthetic polymers lack bioactivity and cell recognition factors, different types of bioactive factors, including growth factors and biologics, can be incorporated into them (Rafique et al., 2021). The second type of biomaterials, designated as natural biomaterials, is derived from natural sources such as polysaccharides and proteins. These biomaterials exhibit high biocompatibility and biodegradability, as well as unique structures that allow the mimicking of the ECM microenvironment. Polysaccharide-based biomaterials include chitosan, alginate, hyaluronic acid, and fibrin, among which chitosan is one of the most widely used cationic polymers used to deliver anionic substances, including growth factors, cytokines, genes, and glycosaminoglycans. Among protein-based biomaterials, including collagen, fibrin, gelatin, and silk, collagen is the most widely used natural biomaterial. Its advantages include simple processing, minimal induction of inflammatory reactions, and approved use by the U.S. Food and Drug Administration (FDA) (7).

Biomaterial-based systems have been exploited to facilitate *in vitro* and *in vivo* tissue repair. Controlled delivery systems for proteins have been used in the instruction of cellular processes, including motility, proliferation, and differentiation (Sneider et al., 2019; Park et al., 2020b; Lee et al., 2021). However, the inherent instability of proteins and their cost are the major limitation for this approach. Under *in vivo* conditions, cell-secreted proteins are sequestered by glycosaminoglycans (GAGs), such as heparin, heparan sulfate, and chondroitin sulfate, which precisely modulate the cellular processes mediated by sequestered proteins (Freudenberg et al., 2016; Hettiaratchi et al., 2016). Positively charged amino acid residues and negatively charged GAGs interact electrostatically, resulting in the sequestration of morphogens and chemokines *in vivo*. This has been mimicked *in vitro* by designing multiple biomaterial platforms capable of sequestering cell-secreted proteins to modulate cell differentiation and metabolism and impact the grafting of transplanted cells for functional tissue repair (Figure 2 and Table 2). Additionally, the preferential sequestration of cytokines and chemokines can be exploited to modulate inflammation and tissue repair processes (Martino et al., 2011; Martino et al., 2014; Lohmann et al., 2017).

**TABLE 2:** Enhancing stem cell function using matrix-anchored bioactive cues.

Biomaterial category	Bioactive cue	Cell/tissue type	Observed effect	References
Heparin-PEG	IGFBP-3, IGFBP-5	ATDC5	Chondrogenic differentiation ↓	Rinker et al. (2018)
Polyelectrolyte multilayered film	SDF-1α	Breast cancer cells	Cell spreading ↓	Picart et al., 2017
PLL/HA				
Soft polymer film	BMP-2	MSCs	Osteogenic differentiation ↑	Picart et al., 2011
			Bone formation ↑	
PMMA-*g*-PEO	EGF	Stromal cells	Cell proliferation ↑	Melarie et al., 2012
			Cell differentiation ↑	
Self-assembling peptide-based hydrogels	IGF-1	MSCs	Stem cell function ↑	Davis et al. (2006)
			Tissue regeneration ↑	
Hydrogel	IFN-γ	hMSCs	Immunomodulatory potential ↑	Gracia et al. (2019)
			Cytokine secretion ↑T cell function ↓	
Hyaluronic acid	N-cadherin mimetic peptide (HAV)	MSCs	Wound healing ↑	Bian et al. (2013)
			Chondrogenesis ↑	
Check polymer	N-cadherin derived peptides	MSCs	Cell signaling ↑	Qin et al. (2020)
			Secretory activities ↑	
Bi-layered PLGA scaffolds	TGF-β1	Islets	MCP-1 ↓	Liu et al. (2016a)
			IL-12 ↓	
			TNF-α ↓	
			Cell engraftment ↑	
Oligoethylene glycol SAM	Heparin-binding peptide	HUVECs, MSCs	Heparin sequestration ↑	Hudalla et al. (2011)
			Cell proliferation ↑	
Hyaluronic acid (HA)-based hydrogels	TGF-β1	CPCs	Proliferation ↑	Jha et al., 2015a and Jha et al., 2015b
			Differentiation ↑	
			VEGF, HGF ↑	

PLL, poly(L-lysine); PLGA, poly(L-lactide-co-glycolide); IGFBP, insulin-like growth factor-binding protein; SDF-1α, stromal cell-derived factor 1 alpha; BMP-2, bone morphogenetic protein-2; EGF, epidermal growth factor; IGF-1, insulin-like growth factor 1; IFN-γ, interferon-gamma; TGF-β, transforming growth factor beta; NPs, nanoparticles; HA, hyaluronic acid; MSCs, mesenchymal stem cells; HUVECs, human umbilical vein endothelial cells; CPCs, cardiac progenitor cells; MCP-1, monocyte chemoattractant protein 1; IL-12, interleukin 12; TNF-α, tumor necrosis factor alpha; VEGF, vascular endothelial growth factor; HGF, hepatocyte growth factor.

Rinker et al. leveraged protein-sequestering heparin sulfate microparticles and successfully sequestrated cell-secreted proteins, which resulted in a reduction in the chondrogenic differentiation of cells *in vitro* (Rinker et al., 2018). Similarly, matrix metalloproteinase (MMP)-degradable hydrogels have been designed to sequester recombinant tissue inhibitors of MMPs (such as rTIMP3) *via* electrostatic interactions. The targeted delivery of rTIMP3-sequestering hydrogels attenuated adverse left ventricular remodeling in a porcine model of myocardial infarction (Purcell et al., 2014).

Using polyelectrolyte films, Liu et al. presented SDF-1α to the ventral side of breast cancer cells, which acted synergistically with CD44 and CXCR4 and impacted cell spreading, as evidenced by the improved formation of lamellipodia and filopodia (Liu et al., 2017b). In contrast, soluble SDF-1α did not affect cellular spreading and migration. Similarly, bone morphogenetic protein-2 (BMP-2) has been locally presented using a soft polymeric film to promote osteogenesis and bone formation by mesenchymal stem cells (MSCs) *in vitro* (Crouzier et al., 2011). It is worth noting that *in vivo* growth factors are present in bound forms in the ECM and are presented to the cells in a spatiotemporal manner (Hettiaratchi et al., 2016). While substantial data exist regarding the modification of polymer scaffolds by ECM proteins and ligands, such as arginine-glycine-aspartic acid (RGD), few studies have discussed the presentation of matrix-bound proteins. Griffith et al. demonstrated that matrix-bound epidermal growth factor (EGF) significantly affects cellular processes (Fan et al., 2007). Recently, matrix-bound growth factors, such as vascular endothelial growth factor (VEGF) and insulin-like growth factor 1 (IGF-1), have been shown to enhance stem cell function and tissue regeneration (Davis et al., 2006; Chen et al., 2010; Shafiq et al., 2018; Wang et al., 2020).

### Investigating Cellular Functions Using Protein-Sequestering Biomaterials

Tethering of cell-instructive biomolecules on biomaterials may assist in the investigating their cellular functions. The immobilization of biomolecules may not only provide the localized presence of cell-instructive cues but also confer enhanced bioactivity to the biomolecules themselves IFN-γ-immobilized hydrogels have been designed to investigate the immunomodulatory function of human mesenchymal stem cells (hMSCs) (Garcia et al., 2019). The tethered form of IFN-γ significantly increases the immunomodulatory potential of hMSCs and elevated the secretion of cytokines to halt the activity of T cells and dendritic cells when compared to the soluble form. The advantages of covalently conjugated IFN-γ are likely related to the increased duration of cell licensing, as the tethered form is present throughout the co-culture period, while the unbound IFN-γ is washed away rapidly. Moreover, the presence of the tethered form of IFN-γ may result in higher local concentrations of IFN-γ surrounding the encapsulated hMSCs than the presence of the unbound form. hMSCs encapsulated within hydrogels with tethered IFN-γ accelerate the healing of colonic mucosal wounds in both immunocompromised and immunocompetent mice (García et al., 2019). However, future studies are needed to optimize the dose of the tethered biomolecules and decipher the cellular and biological cascades involved so that the efficacy of the therapy can be increased.

It is noteworthy that while *ex vivo* licensing of hMSCs is therapeutically effective, significant technical, regulatory, and economic issues limit the translational potential of this approach. However, the biomaterial-assisted licensing of hMSCs may greatly enhance their immunomodulatory potential. Although extensive research has been conducted to show biomaterial-assisted tissue regeneration, biomaterials have rarely been exploited for *ex vivo* licensing of stem cells (Bian et al., 2013; Cosgrove et al., 2016; Li et al., 2017; Zhang et al., 2018b).

To mimic the *in vivo* microenvironment and promote the chondrogenic differentiation of MSCs *in vitro* and *in vivo*, Bian et al. engineered HA hydrogels containing N-cadherin mimetic peptides (Bian et al., 2013). In particular, the His-Ala-Val (HAV) motif present in the first extracellular domain (ECD1) of N-cadherin, which is critical for the homotypic protein interactions that mediate cell-cell adhesion, was engineered into the HA matrix. The tethered HAV motif promoted not only the early chondrogenic differentiation of MSCs and cartilage-specific matrix production *in vitro*, but also neocartilage formation in implants functionalized with N-cadherin mimetic peptides after subcutaneous implantation. Leckband et al. also evaluated the potential of the full N-cadherin protein, as well as the first two ectodomains of N-cadherin, and a peptide containing the histidine-alanine-valine-aspartic acid-valine (HAVDI) sequence in the first extracellular domain (Qin et al., 2020). While MSCs exhibited comparable rigidity-dependent spreading and traction forces, they differed in cell signaling and secretory activities.

The local presentation of bioactive cues at the implantation site may also reduce the inflammatory response by lowering the expression of inflammatory cytokines, thereby reducing graft rejection. In addition, they can be used to investigate the function of encapsulated cells. Liu et al. encapsulated TGF-β into a bi-layer scaffold, which not only reduced the expression of proinflammatory cytokines, such as monocyte chemoattractant protein 1 (MCP-1), interleukin-12 (IL-12), and tumor necrosis factor-alpha (TNF-α), but also improved the survival and engraftment of transplanted islet cell*s in vivo* (Liu et al., 2016a). Importantly, TGF-β-containing scaffolds showed improved glucose levels compared to the control group.

Hudalla et al. conjugated heparin-binding peptide with oligoethylene glycol self-assembling monolayers and reported that the tethered peptide can bind heparin *in vitro*, thereby sequestering heparin-binding growth factors (Hudalla et al., 2011). Notably, sequestered heparin-bound fibroblast growth factor 2 (FGF-2) enhanced the proliferation of human umbilical vein endothelial cells (HUVECs) and MSCs *in vitro*. It is expected that such a strategy could also enhance the sequestration of MSC-secreted factors *in vivo*. Likewise, Heally et al. recently designed cross-linked HA-based hydrogels containing covalently crosslinked heparin and TGF-β1, which significantly increased the proliferation, differentiation, and formation of tubular structures in encapsulated cardiac progenitor cells (CPCs) (Jha et al., 2015a; Jha et al., 2015b). Additionally, the levels of several matrix-bound morphogens which significantly contribute to endothelial cell mobilization and vascular invasion, such as insulin-like growth factor-binding proteins (IGFBPs), VEGF, and hepatocyte growth factor (HGF), were significantly increased. This suggests that heparin presentation concentrated and amplified cell-secreted signals to enhance CPC maturation.

## Dynamic Biomaterial-Mediated Stem Cell Fate Modulation

### Mechanobiology: A Tool for Cellular Mechanosensing

It has been widely accepted that cells respond to the ECM via mechanosensing, which drives their migration, proliferation, and differentiation (Discher et al., 2005; Wozniak and Chen, 2009). A myriad of substrates with varying physical properties, such as stiffness, elasticity, and modulus, have been investigated to study the effect of physical parameters on cellular behavior via cellular mechanotransduction. It has been demonstrated that scaffolds with varying stiffness and moduli of elasticity impact cellular behavior differently. Moreover, it has been observed that an increase in the stiffness of cell culture platforms based on collagen-coated polyacrylamide affects the migration, neuronal differentiation, spreading, and stemness of the cells (Pelham and Wang, 1997; Engler et al., 2004; Gilbert et al., 2010).

Despite significant progress in understanding the role of matrix mechanics and elasticity in cellular processes and cellular mechanotransduction, much remains to be investigated. Scientists have discovered new molecules involved in cellular mechanotransduction and have decoupled the effects of material viscoelasticity and viscoplasticity on cellular processes (Table 3) (Marozas et al., 2019). The ECM is not purely elastic, but it also exhibits viscoelastic characteristics; therefore, careful elucidation of both the elastic and viscoelastic components of the ECM in cellular processes is needed (Fung, 1993; McKinnon et al., 2014a; Chaudhuri et al., 2016; Rosales and Anseth, 2016). The distinct role of the viscoelasticity of the matrix on cellular behavior has only recently been studied. While elastic materials store energy, viscoelastic materials can dissipate applied stresses and may undergo stress relaxation in response to deformation and creep in response to them.

**TABLE 3:** Enhancing stem cell function using instructive biomaterials and mechanotransduction.

Biomaterial	Cell/tissue type	Observed effect	References
Collagen-based hydrogels	MSCs	Osteogenic differentiation ↑	Lou et al. (2018)
Hydrogels with tunable dissociation rate constant	MSCs	Spreading ↑	Yang et al. (2021)
		Osteogenic differentiation ↑	
Hydrogels	MSCs	alpha-SMA marker ↑	Cameron et al. (2011)
		Calponin ↑	
Alginate-based hydrogels	hMSCs	Osteogenic differentiation ↑	Darnell and Mooney, (2017)
		Biomineralization ↑	
		Matrix secretion ↑	
		Cell retention ↑	
RGD-PEG-PLA nanoparticles	hMSCs	Cell retention Cell viability ↑	Grosskopf et al. (2020)
		Cell engraftment ↑	
HA-based hydrogels	ADSCs	Cell retention ↑	Andreina Parisi-Amc et al., 2013
PINIPAM-C7-based hydrogels containing YIGSR, IKVAV	Schwann cells	Cell retention ↑	Marquardt et al. (2020)
PEG-based hydrogels	Chondrocytes	ECM secretion ↑	Richardson et al. (2019)
		GAG secretion ↑	Feng et al. (2019)

PINIPAM, poly(N-isopropyl acrylamide); HA, hyaluronic acid; MSCs, mesenchymal stem cells; YIGSR, (Tyr-Ile-Gly-Ser-Arg); IKVAV, (Ile-Lys-Val-Ala-Val); ECM, extracellular matrix; GAGs, glycosaminoglycans; α-SMA, alpha-smooth muscle actin; PEG, poly(ethylene glycol); hMSCs, human mesenchymal stem cells.

Consequently, viscoelastic materials possess elastic and viscous components that affect cellular behavior differently. More specifically, viscoelastic materials undergo two important transitions: contraction in response to applied stress and viscoelastic relaxation when a constant strain is applied. Evaluation of the mechanical properties of most tissues, including brain, adipose, liver, fibrin clot, and fractured hematoma, have shown that they exhibit viscoelastic characteristics (Fung, 1993; McKinnon et al., 2014a; Chaudhuri et al., 2016; Rosales and Anseth, 2016). Consequently, researchers have recently developed biomaterials with varying degrees of viscoelasticity and demonstrated the significant impact of viscoelasticity on cellular behaviors (Chaudhuri et al., 2016; Rosales and Anseth, 2016).

### Engineering Biomimetic Platforms for Cellular Mechanosensing

It is generally understood that in a two-dimensional (2D) microenvironment, cells bind to the matrix through integrins and exert force on it; reciprocally, cells gauge the matrix stiffness *via* cellular machineries, such as the actin cytoskeleton and molecules that interact with it, such as vinculin and talin. As viscoelastic hydrogels possess stress relaxation and viscoelastic creep, which may dissipate the applied force, cells may, in turn, perceive less stiffness, thereby spreading less. In contrast, *in vitro* cell culture on 2D collagen-coated polyacrylamide hydrogels resulted in enhanced spreading of MSCs with an increase in the loss modulus or creep (Cameron et al., 2011). Similar results have been found for alginate hydrogels, wherein cells spread more in soft hydrogels than in stiff hydrogels (Chaudhuri et al., 2015). One possible explanation for this observation may be due to increased stability of integrin-ECM bonds that behave as slip bonds owing to stress relaxation. This is because a decrease in the individual bond force leads to an increase in bond lifetime (Chan and Odde, 2008).

The impact of the viscoelasticity of the hydrogels was assessed using 3D cell culture. Just as 2D cell culture influences cellular behavior, 3D culture also significantly impacts cell spreading, proliferation, and differentiation (Das et al., 2016). While synthetic hydrogels are nanoporous, which perturbs cell expansion or volume change during spreading, installing degradable crosslinks in hydrogels partly overcomes this issue. In addition, viscoelastic hydrogels permit cell expansion and volume change (Khetan et al., 2013). Moreover, it has been demonstrated that lowering the stress relaxation time significantly impacts cell spreading (McKinnon et al., 2014a; McKinnon et al., 2014b). Likewise, a reduction in the stress relaxation time promoted the spreading of fibroblasts in RGD-coupled alginate hydrogels (Chaudhuri et al., 2015).

As most cell culture platforms include ECM-derived proteins, such as collagen, gelatin, and Matrigel, the manifested cellular behavior could be partly ascribed to viscoelasticity of these platforms (Nam et al., 2016a; Nam et al., 2016b). Lou et al. demonstrated that interpenetrating hydrogel systems based on HA-crosslinked with dynamic covalent bonds and collagen favored cell spreading and fiber remodeling. More importantly, while most conventional hydrogels are bio-inert, collagen-based hydrogels are not only biocompatible but also recapitulate the fibrillar network, as observed in the ECM microenvironment (Lou et al., 2018). Hydrogels containing more crosslinks promote osteogenic differentiation of the encapsulated cells. Bian et al. demonstrated better spreading and osteogenic differentiation of encapsulated cells in hydrogels with large dissociation rate constants than those of hydrogels with low dissociation rate constants, which was dependent on the concerted action of the cell adhesion structures containing β1 integrins (Yang et al., 2021). This indicates that the biofunctionalization of hydrogels also crucial for realizing ECM mimetic effects.

### Investigating Cell Fate Using Mechanocompatible Materials

Varying degrees of stress relaxation and creep have also been reported to affect cellular differentiation. Cameron et al. reported that MSCs cultured on high-creeping hydrogels differentiated into smooth muscle cells (SMCs), which largely expressed SMC markers, such as alpha-smooth muscle actin (α-SMA) and calponin, compared to cells seeded on low-creep hydrogels. In the presence of induction factors, hMSCs cultured on high-creep hydrogels also expressed multiple differentiation capabilities compared to those cultured on low-creep hydrogels (Cameron et al., 2011). It was further demonstrated that hMSCs compensate for decreased passive cytoskeletal tension by spreading via activation of Ras-related C3 botulinum toxin substrate 1 (Rac1), which is a mechanotransductive Rho-GTPase that also plays a role in SMC differentiation.

Mooney et al. also reported the effect of stress relaxation on hMSC differentiation and found that the cells cultured on fast-relaxing alginate-based hydrogels were committed toward the osteogenic lineage *in vitro*, along with showing higher biomineralization and more matrix secretion than those cultured on slow-relaxing hydrogels (Darnell and Mooney, 2017). More importantly, the implantation of the fast-relaxing hydrogels along with hMSCs led to more bone formation *in vivo* than that of the control group. Defects implanted with fast-relaxing hydrogels were also filled faster than those implanted with slow-relaxing hydrogels. Examination of the remaining hydrogels in the defect at 2 weeks after implantation revealed that only a small amount of alginate remained in the groups treated with the fast-relaxing hydrogels compared to that of the defects treated with the slow-relaxing hydrogels, which was ascribed to the cellular remodeling of the biomaterials *in vivo*. These findings encourage further evaluation of stress-relaxing biomaterials for *in vivo* studies.

### Enhancing Stem Cell Survival and Retention Using Mechanocompatible Biomaterials

**FIGURE 3:**
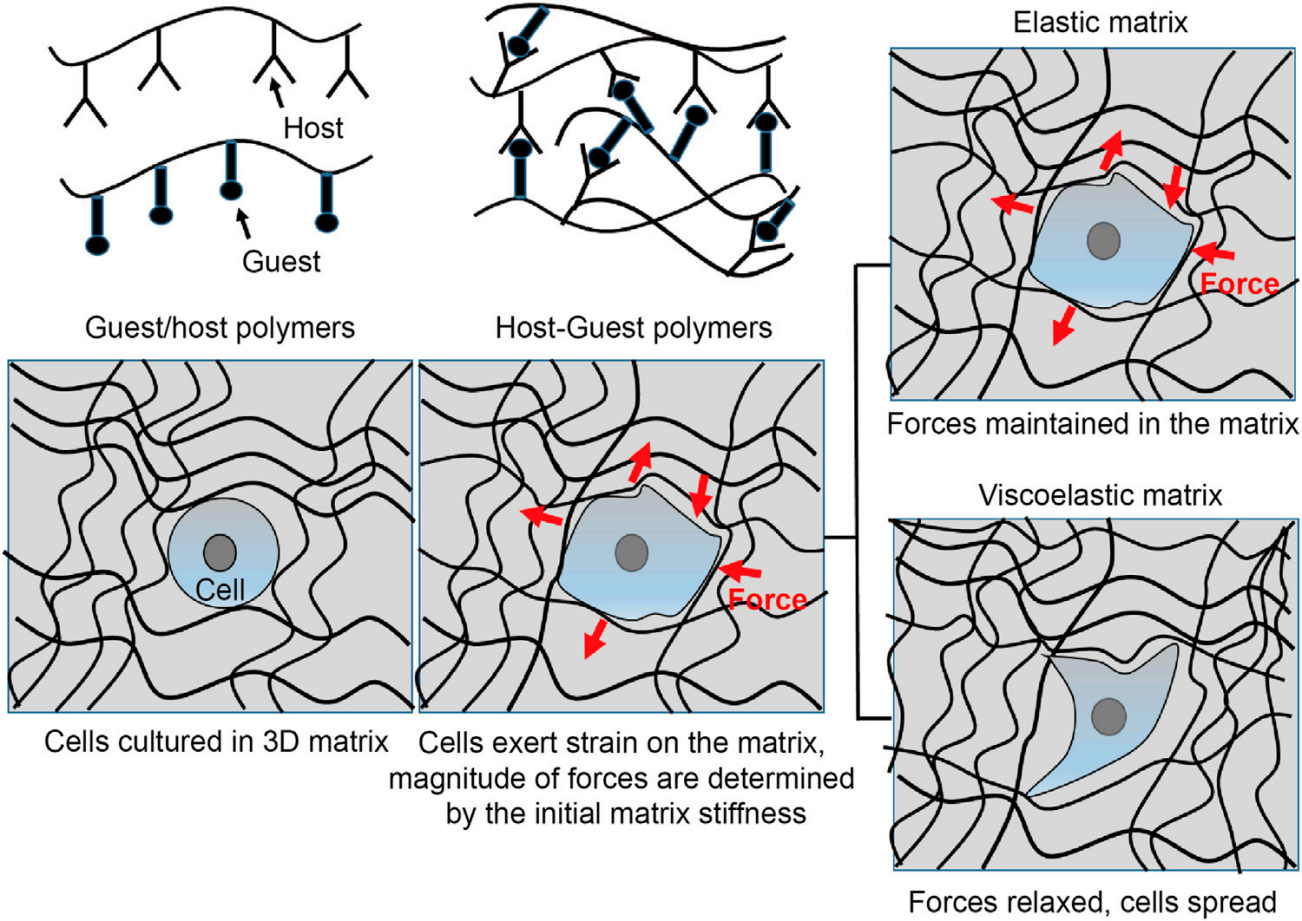
Interrogating cell function by mechanosensing. Non-covalently crosslinked biomaterials can be engineered to interrogate cell fate by mechanotransduction. Such materials can be designed by installing functional moieties on the individual polymer chains to provide shear-thinning and self-healing characteristics. Once cultured in the elastic or viscoelastic cell substrates, cells can experience different mechanotransduction. In a purely elastic substrate, the cells probe the matrix via integrins and possess spherical shape. On the other hand, if they are cultured on a viscoelastic substrate, both the elastic and viscoelastic components of the matrix can regulate the cell function. Consequently, the cell-mediated forces can be relieved or dissipate in the viscoelastic matrix. The cells can also deform the matrix during spreading, proliferation, and migration.

Shear-thinning and dynamic hydrogels have enabled long-term survival and retention of transplanted cells *in vivo*, which is partly attributable to the lesser force experienced by the cells during the injection and the conducive environment provided by the physically or non-covalently crosslinked hydrogels (Figure 3). The Appel group has developed supramolecular injectable polymer nanoparticle-based hydrogels containing RGD-tethered polyethylene glycol-poly(L-lactide) (RGD-PEG-PLA), which exhibited shear-thinning and self-healing and significantly improved cell retention as compared to the phosphate buffered saline (PBS) control after subcutaneous implantation *in vivo* for up to 2 weeks (Grosskopf et al., 2020). Likewise, Heilshorn et al. reported significant retention of transplanted adipose-derived stem cells (ADSCs) using shear-thinning and self-healing protein-based hydrogels as assessed using bioluminescent imaging (BLI) *in vivo* for up to 2 weeks (Parisi-Amon et al., 2013). The beneficial effect of the engineered protein-based hydrogels as compared to collagen and alginate is due to the protection of cells during transplantation, instantaneous formation of gels during the injection process, and cell signaling activated by the RGD ligand and hydrogels after transplantation *in vivo*.

Heilshorn et al. also designed a new variant of shear-thinning and self-healing hydrogels, in which laminin-derived peptide moieties, including pentapeptide, Tyr-Ile-Gly-Ser-Arg (YIGSR), and Ile-Lys-Val-Ala-Val (IKVAV), were incorporated into the hydrogels. They engineered protein-based supramolecular hydrogels by exploiting weak peptide-peptide interactions between the recombinantly engineered C7 protein and the poly(N-isopropylacrylamide) (PNIPAM) and proline-rich peptide-tethered star-shaped PEG-based hydrogels. Once exploited for the transplantation of Schwann cells (SCs) in a spinal cord injury (SCI) model, these hydrogels remarkably improved cell retention *in vivo* (740% higher cell retention vs. saline group) (Marquardt et al., 2020). The enhanced retention of transplanted SCs also led to functional recovery *in vivo*. Consequently, these hydrogels exhibit great potential for applications in cell biology and regenerative medicine (Wang et al., 2009). Anseth et al. reported significant secretion of ECM proteins and GAGs in PEG-based hydrogels crosslinked by hydrazone bonds (Richardson et al., 2019). At 4 weeks post-encapsulation, the experimental chondrocytes secreted 190 ± 30% more collagen and 140 ± 20% more sulfated GAGs than those in the control group, which mainly constrained matrix deposition only to the pericellular space.

The same group also investigated the chondroprotective effect of the adaptable hydrazone-crosslinked hydrogels, which displayed a high number of cartilage-specific markers, such as SOX9, and led to the increased deposition of collagen and GAGs. These hydrogels demonstrated an average of 70 ± 4 µg of sulfated GAGs per day and 31 ± 3 µg of collagen per day over 1 month in dynamic compression bioreactors. These types of hydrogels can also be further strengthened by chemical bonds to realize controllable drug release, cellular infiltration, and matrix deposition (Feng et al., 2019). These findings highlight how adaptable hydrogels can enhance regenerative capability, which may have wider implications for regenerative medicine and tissue engineering applications.

## Enhancing Stem Cell Retention and Engraftment for a Functional Tissue Repair

### Strategies to Enhance Stem Cell Survival and Retention *in vivo*

**FIGURE 4:**
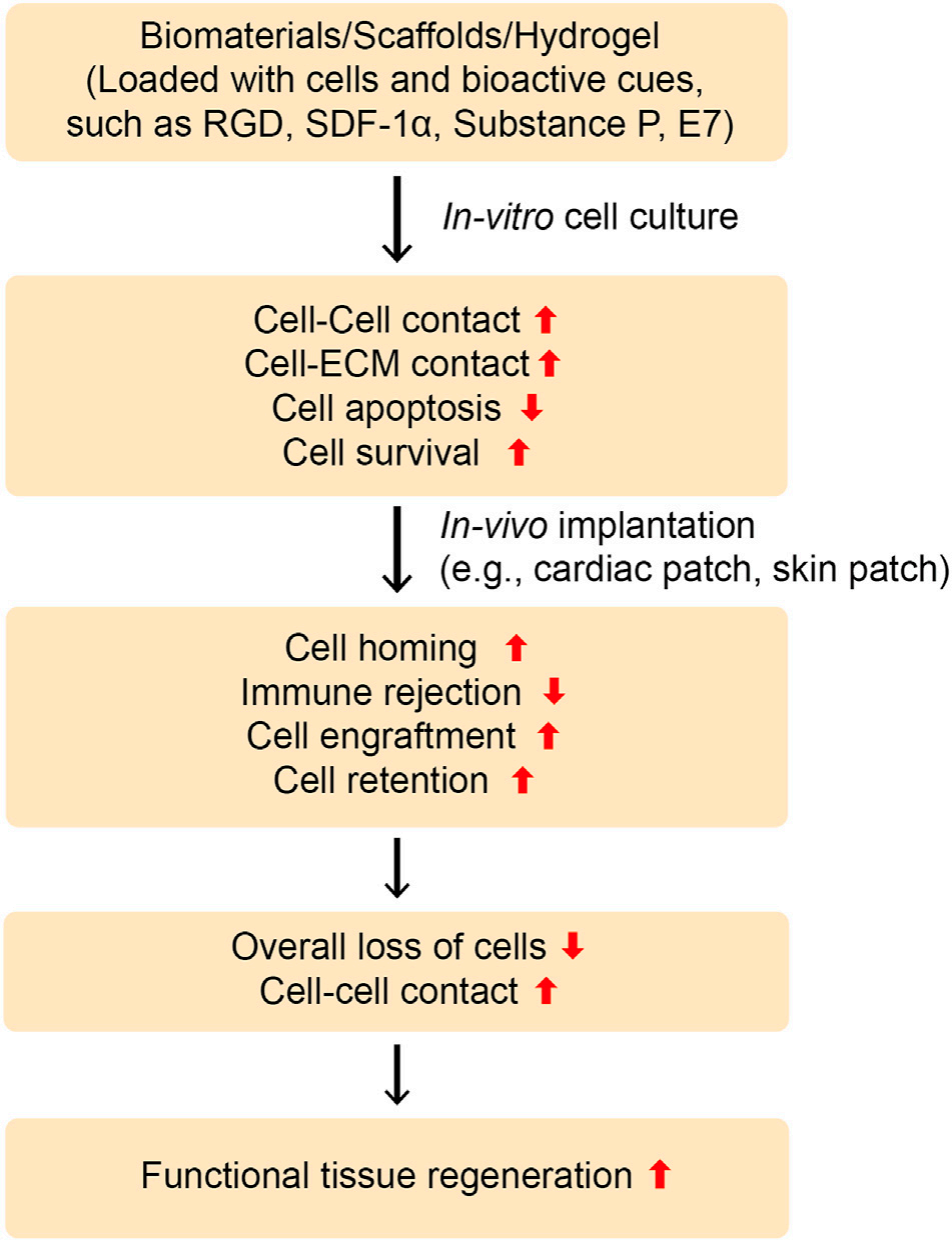
Designing instructive biomaterials for tissue regeneration applications. To ameliorate cell-cell and cell-ECM contacts and impact cellular behavior, instructive biomaterials can be designed by installing an array of functional moieties on biomaterials, such as cell-adhesive ligands like RGD or laminin-derived bioactive cues (IKVAV or YIGSR), the MSCs-affinity peptide “*E7*”, or the EPCs-capturing peptide “*TPS*”. Furthermore, cell inducing/recruiting factors can be incorporated into biomaterials to improve stem cell mobilization, homing, and recruitment *in vitro* and *in vivo*. Various cell inducing and recruiting factors, including SDF-1α and SP have been integrated into biomaterials and shown to promote cell homing and migration. These bioactive cues could be central to ameliorate cell-cell and cell-ECM contacts as well as improve cell survival and retention during tissue regeneration *in vivo*. Overall, this can lead to functional tissue repair.

**FIGURE 5:**
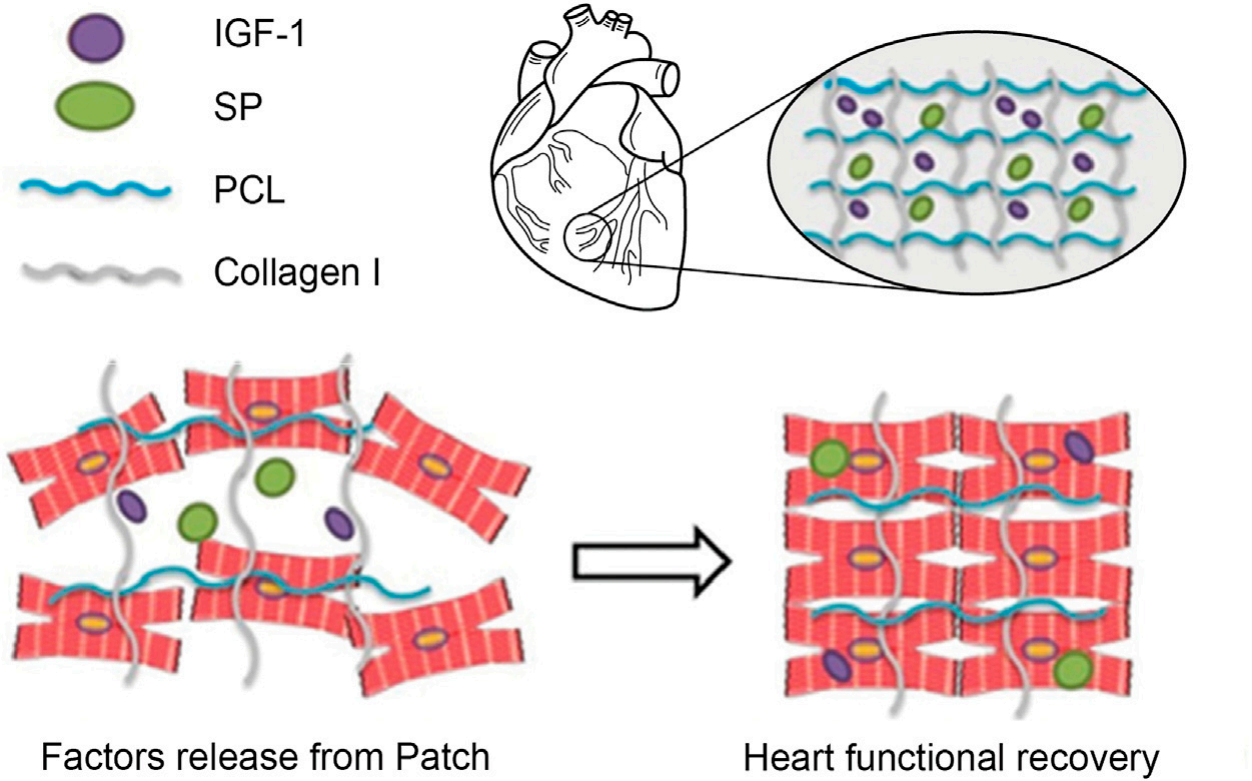
Schematic illustration of MSCs Recruitment in vivo. Schematic diagram of the designed study. Cardiac patches containing SP alone or SP and IGF-1 peptides were fabricated by electrospinning. SP may recruit endogenous stem and progenitor cells, whereas IGF-1 peptide may enhance their retention and engraftment at the target site. Adapted with permission from (Shafiq et al., 2018). Copyrights reserved Oxford Publishers.

While MSCs exhibit enormous potential for tissue repair due to their multilineage commitment, their use in clinical settings is hampered partly due to their poor homing to the target site and is further compromised by their poor retention and engraftment at the target site. Therefore, enhancing stem cell homing to the target site as well as improving retention and engraftment may improve the clinical translatability of stem cell therapy. Stem cell transplantation using conventional techniques, such as intravascular or intracardiac injection, leads to the loss of majority of cells, primarily due to poor cell-cell and cell-ECM interactions (Ullah et al., 2015; Lee et al., 2021). Moreover, most of the transplanted cells may lose their immunomodulatory potential. Taken together, these results show the necessity of either preconditioning and licensing therapeutic cells before transplantation or using vehicles for stem cell transplantation (Park et al., 2018). In this section, we discuss recent techniques that have been leveraged to enhance stem cell transplantation, including homing, retention, and engraftment (Figures 4, 5, Table 4).

**TABLE 4:** Enhancing stem cell retention using instructive biomaterials.

Biomaterial	Bioactive cue	Cell/tissue type	Observed effect	References
PLCL/heparin nanofibers	SDF-1α		c-kit positive cells ↑	Shafiq et al. (2017)
			BMSCs ↑	
			Blood vessels ↑	
Microbubbles	SDF-1α	BMSCs	CXCR4 ↑	Xiang et al. (2020)
			Cell migration ↑	
PLGA/HA NPs	SDF-1α	MSCs	SDF-1α release ↑	Teo et al. (2020)
			CXCR4 ↑	
			Transendothelial cell migration ↑	
			Biodistribution ↑	
PLGA-HA NPs	EGCG, MnO_2_ nanocatalysts	MSCs	Metabolic potential ↑	Teo et al. (2020)
			Angiogenesis ↑	
Octadecylamine-grafted HA-encapsulating liposomes	TNF-α-encapsulating liposomes	ADSCs	VEGF ↑	Leong et al. (2020)
			PGE2 ↑	
			Vascularization ↑ Pigment epithelium-derived growth factor ↑	
PCL scaffolds	GDNF, BDNF	NSCs	Perfusion ↑	Wang et al. (2016)
			Cell survival ↑	
			Cell proliferation ↑	Nisbet et al. (2018)
			Neurites outgrowth ↑	
SAP-based hydrogels	GFs		Astroglia ↓	Bruggeman et al. (2017)
			Growth factors release ↑	
APICLS	Paracrine factors	MSCs	Angiogenesis ↑	Park et al. (2018)
			Cardioprotection ↑	
			Contractile forces ↑	
			Cardiac remodeling ↓	Park et al. (2020a)
			Fibrosis ↑	
Decellularized omentum tissues	iPSCs-CMs	MSCs	Cell survival ↑	Gal et al., 2020
			Cell retention ↑	
PPZ-based hydrogels	TGF-β-derived peptides	MSCs	Cell survival ↑	Hong et al. (2019)
	CESPLKRQ & HAV		Chondrogenesis ↑	
Oxygen-preserving hydrogels	HEMA	BMSCs	Cell survival ↑	Niu et al. (2020)
			Cell proliferation dsDNA content of cells ↑	
PCL/Col-1 nanofibers	SP, IGF-1C	-	Cell mobilization ↑	Shafiq et al. (2018)
			Vascularization ↑	
			Cardiac function ↑	

PLCL, poly(L-lactide-co-caprolactone); PLGA, poly(L-lactide-co-glycolide); PCL, polycaprolactone; Col-1, collagen type 1; HA, hyaluronic acid; NPs, nanoparticles; SAP, self-assembling peptides; PPZ, poly(organophosphazene); SDF-1α, stromal cell-derived factor 1 alpha; BDNF, brain-derived neurotrophic factor; GDNF, glial-derived neurotrophic factor; iPSC-CMs, induced pluripotent stem cell-derived cardiomyocytes; TGF-β, transforming growth factor-beta; MSCs, mesenchymal stem cells; BMSCs, bone marrow mesenchymal stem cells: HEMA, 2-hydroxyethyl methacrylate.

While transplanted stem cells display poor homing to the target site, certain cytokines and chemokines have been implicated in improving cell homing and could be used to overcome this problem. For instance, SDF-1α has been demonstrated to improve stem cell mobilization, activation, homing, and retention in multiple tissues (Xiang et al., 2020). The SDF-1α-CXCR4 axis has been reported to play a vital role in SDF-1α-mediated cell migration and homing (Petit et al., 2007; Marquez-Curtis and Janowska-Wieczorek, 2013; Shafiq et al., 2017). While hematopoietic stem cells (HSCs) actively express CXCR4, which helps SDF-1α-mediated homing to the bone marrow (BM) as well as transendothelial migration, only a small proportion of MSCs express CXCR4. Consequently, different strategies have been proposed to elevate the expression of CXCR4 in MSCs to improve their trafficking *in vitro* and *in vivo* (Teo et al., 2020; Xiang et al., 2020)*.* Xiang et al. leveraged chemotactic microbubble-conjugating SDF-1α, which significantly enhanced the expression of CXCR4 in bone marrow mesenchymal stem cells (BMSCs) during *in vitro* culture and improved their migration *in vitro* and *in vivo* after systematic transplantation in an osteoarthritis rat model (Xiang et al., 2020).

### Instructive Biomaterials to Enhance Stem Cell Function *in vivo*

Kong et al. designed poly(D, L-lactide-co-glycolide) (PLGA)-conjugated HA (PLGA-HA) nanoparticles and encapsulated recombinant SDF-1α protein. PLGA-HA nanoparticles anchored onto MSCs and sustainably released SDF-1α, resulting in an elevated expression of CXCR4 (a 7.0-fold increase compared with untreated MSCs) (Teo et al., 2020). Nanoparticle-mediated release of SDF-1α also improved transendothelial migration *in vitro* and biodistribution in a hindlimb ischemia model *in vivo*. Altogether, these results indicate the potential of the SDF-1α-CXCR4 axis in stem cell homing. The same group also leveraged PLGA-HA nanoparticles encapsulating antioxidative cargos (epigallocatechin gallate, EGCG) and manganese oxide (MnO_2_) nanocatalysts) to improve the survival of MSCs in the oxidative tissue microenvironment, which conferred better metabolic and angiogenic potential on the cells (Teo et al., 2020). Similarly, to impact the secretory profile of ADSCs, octadecylamine-grafted HA was complexed onto a liposomal carrier of TNFα, which upregulated the secretion of proangiogenic VEGF and immunomodulatory prostaglandin E2 (PGE2) while decreasing the secretion of antiangiogenic pigment epithelium-derived factors (Leong et al., 2020). TNF-α-tethered ADSCs also promoted vascularization on a chip *in vitro* as well as perfusion in a murine ischemic hindlimb model *in vivo*.

Similarly, Parish et al. installed glial cell-derived neurotrophic factor (GDNF) and brain-derived neurotrophic factor (BDNF) on electrospun PCL-based scaffolds to enhance the survival, retention, and engraftment of transplanted neural stem cells (NSCs) (Wang et al., 2016; Nisbet et al., 2018). Both *in vitro* and *in vivo* results showed that the covalently immobilized growth factors enhanced the survival of transplanted NSCs into the targeted tissues. Additionally, GDNF-immobilized scaffolds improved the proliferation and differentiation of NSCs and neurite outgrowth of the transplanted cortical cells while suppressing the number of inflammatory reactive astroglial cells. Nisbet et al. recently embarked on a combination approach for growth factor delivery by blending tissue-specific self-assembling peptide (SAP)-based hydrogels and short nanofibers (Bruggeman et al., 2017). While SAP-based hydrogels provided the rapid release of growth factors, the release started from nanofibers after 6 days in a sustained fashion. These fiber-reinforced hydrogels can be exploited for growth factor delivery and stem cell-mediated tissue repair. Somaa et al. also leveraged brain ECM-mimicking SAP-based hydrogels expressing the laminin-derived epitope “IKVAV” to improve the transplantation of embryonic stem cell (ESC)-derived cortical progenitors (Somaa et al., 2017). A network of green fluorescent protein (GFP) was observed in the subcortical nuclei, including the hippocampus, lateral septum, and striatum, as well as in the ventral thalamus and hypothalamus. The cell-seeded scaffolds showed potential to improve neuronal function and led to an improvement in motor function over 9 months as compared to the cell- or scaffold-only group (Lee et al., 2018). Cha et al. introduced adhesive protein-based immiscible condensed liquid systems (APICLS) to enhance the survival, retention, and integration of cells with the target tissues (Park et al., 2020c). The APICLS consisted of an immiscible modality from sandcastle worms and an adhesive modality from mussels. Fluid immiscibility may result in the enhanced integration of hydrogels with the myocardium, while adhesiveness may improve the integration of transplanted cells with the host myocardium, concomitantly resulting in enhanced retention and engraftment of the transplanted cells. The hydrogels integrated well with the host myocardium and increased the persistence of the transplanted cells. Secreted paracrine factors released from APICLS with MSCs induced angiogenesis and cardioprotection, and delayed cardiac remodeling, reduced fibrosis, and allowed the recovery of contractive force.

As the loss of cell-cell and cell-ECM interactions leads to reduced survival of transplanted cells, biomaterial-assisted transplantation has been widely pursued to overcome these limitations (Han et al., 2020; Jo et al., 2020). Most importantly, hydrogels fabricated from natural and synthetic polymers have been proposed to improve stem cell transplantation and engraftment into target tissues. Recently, decellularized ECM-based hydrogels have shown promising results compared to conventional hydrogels. For instance, Dvir et al. decellularized omentum tissues and created thermoresponsive omentum ECM-derived hydrogels that induced pluripotent stem cell (iPSC)-derived cardiomyocytes (CMs) (Shevach et al., 2015). CMs were encapsulated in ECM-derived hydrogels using microfluidics. Once injected into the mouse gastrocnemius muscle, these cellular microdroplets showed good survival and retention for up to 2 days.

However, the long-term fate of transplanted cells remains to be elucidated. Successful cell-mediated tissue repair requires the concerted contribution of stem cell transplantation, stem cell viability at the lesion site, and stem cell differentiation into the targeted lineages. Simultaneously enhancing stem cell transplantation and improving the viability and differentiation of the transplanted cells holds great promise for functional tissue repair. This has been realized, at least in soft and hard tissue regeneration. For instance, exogenously transplanted or endogenously recruited stem cells have been directed to differentiate into the targeted lineage, such as osteocytes or chondrocytes, by incorporating chondrogenic growth factors (TGF-β3) or BMP-2 (Noh et al., 2015; Lee et al., 2017). Similarly, in a seminal study, Song et al. designed poly(organophosphazene) (PPZ)-based thermosensitive hydrogels and incorporated a TGF-β1-derived peptide sequence (CESPLKRQ) and N-cadherin-derived peptide sequences containing elongated HAV sequences such as CLRAHAVDIN (Hong et al., 2019). More importantly, these chondrogenic peptides were incorporated into the hydrogels by exploiting host-guest interactions by installing β-cyclodextrin (β-CD) on (PPZ) and deriving peptides with adamantane (host). The engineered hydrogels improved stem cell transplantation owing to their injectability and extended viability of the transplanted MSCs, as revealed by the *in vivo* BLI. The tethered peptide moieties promoted chondrogenesis of transplanted MSCs through mitogen-activated protein kinase (MAPK) pathways. These results suggest that simultaneous approaches can be used to realize MSC-mediated therapeutic benefits.

As most of the transplanted cells are lost due to hypoxia, transplantable cell types are either treated by hypoxic preconditioning or by using oxygen generated *in situ*. However, stem cell preconditioning as well as the *in situ* generation of oxygen may not be suitable in many cases because it can result in the overproduction of hydrogen peroxide (H_2_O_2_), which may be disadvantageous or cytotoxic to healthy cells. The general method is to incorporate calcium oxide (CaO_2_) or magnesium oxide (MgO_2_) into the tissue culture or hydrogel, which may then react with water to produce H_2_O_2_. The decomposition of H_2_O_2_ yields oxygen. The modification of biomaterials to supply sufficient amounts of oxygen could be a viable option to address the loss of transplanted cells by hypoxia. Guan et al. designed oxygen-preserving hydrogels that enhanced the survival of BMSCs for up to 14 days in an *in vitro* culture. In contrast, extensive cell death was observed in the control group (Niu et al., 2020).

### Instructive Biomaterials to Enhance Function of Extracellular Vesicles

While a myriad of cell types, including stem cells and pluripotent stem cells, have been demonstrated to improve tissue repair, limited cell retention and engraftment at the target site hampers cell therapy. Accordingly, a multitude of biomaterials have been researched to ameliorate the limitations associated with cell therapy, and this has proven to be an effective approach. Stem cell-associated amelioration of the injury microenvironment is mediated by paracrine mechanisms, including the secretion of trophic factors. Because they contain paracrine factors as well as harboring mRNAs and miRNAs, these cell-secreted EVs hold great promise for regenerative medicine and tissue engineering, and have also been used as carriers for drug delivery and tissue repair.

To further expand their use, different types of biomaterial-centered approaches have been proposed to improve the quantity and content of EVs or augment their delivery and retention into target tissue types (Paganini et al., 2019; Iavorovschi and Wang, 2020). For instance, positively charged superparamagnetic iron oxide nanoparticles (SPIONs) encapsulating PLGA-PEI nanoparticles have been shown to cause a significant improvement in the yield of EVs, which was mediated by the autophagy-stimulated *Beclin-1* gene (Park et al., 2020a). In addition, exploited bioreactor-mediated mechanical forces improved the yield of exosomes, which was mediated by the mechanosensitivity of yes-associated protein (YAP) mechanosensitivity (Guo et al., 2021). Likewise, the differential response of periodontal stem cell-derived EVs upon culture in three-dimensional magnetically strained culture and, more interestingly, those derived from the strained culture also exhibited better bioactivity *in vitro* and *in vivo* when compared to those in a 3D culture microenvironment (Yu et al., 2021). Enhanced expression of miR-133-3p in macrophage migration inhibitory factor (MIF)-engineered umbilical cord MSC (ucMSC)-derived EVs was observed compared to the control umMSCs group (Zhu et al., 2021). Similarly, stimulating human placental-derived MSCs using NO-releasing polymers has been shown to have a significantly improved angiogenic effect on HUVECs, mediated by an increased expression of VEGF and miR-126. However, due to overlapping size range and the lack of specific markers, current EVs preparations, including exosomes are highly heterogenous with undefined purity. International Society for Extracellular Vesicles (ISEV) has introduced guidelines/recommendations for the minimal information for studies of EVs (MISEV) regarding the nomenclature, isolation, and characterization of EVs, which outlines an exhaustive platform for EV related research (Thery et al., 2018).

In addition to improving the quantity and content of EVs, enhancing their retention and sustained release at the injury site also holds great promise, which has been addressed by exploiting different types of strategies, including immobilization on scaffolds or encapsulation into hydrogels. For instance, Li et al. leveraged integrin-ligand interactions to immobilize MSC-derived EVs on HA-based hydrogels for degenerated nerve regeneration, and the immobilized EVs exhibited greater retention and sustained release into nerve tissues. This not only improved motor function and urinary tissue preservation but also mitigated inflammation and oxidation (Li et al., 2020). Because the majority of intravenously administered EVs are adsorbed within the liver, biomaterial-mediated strategies can be exploited to improve their homing to the target tissues. Recently, EVs covalently conjugated with cardiac homing peptide (CHP, CSTSMLKAC) have shown a considerably improved homing effect to infarcted myocardium, which resulted in improved cardiac function and reduced fibrosis (Vandergriff et al., 2018).

Chemical immobilization of the targeting ligand is an interesting avenue, but harsh chemical conjugation chemistries can hamper this approach. Biorthogonal labeling of EVs during their secretion from the cell in the presence of azide-containing moieties followed by alkyne-azide click conjugation of target proteins, such as biotin, improved cellular uptake of EVs as well as the delivery of target loads into cells (Wang et al., 2015). EVs with magnetoresponsive ability have been further developed by employing oleic-acid modified iron oxide nanoparticles, which exhibit magnetic field-mediated homing to adipose tissue-derived stem cells (ADSCs) (Mizuta et al., 2019). To further leverage EVs with the potential to evade macrophage uptake and improve their cellular internalization, the platelet membrane may be anchored onto EVs. Platelet membrane hybridized EVs have exhibited macropinocytosis-mediated cellular internalization as well as distinct myocardial homing compared to unmodified EVs (Hu et al., 2021).

In addition to their modification, polymeric carriers have been designed to allow the controlled release of EVs. For instance, a PLGA copolymer was used to deliver odontogenic EVs, which helped to regenerate dentin (Swanson et al., 2020) and the introduction of phage-display derived “LLP2A” ligand capable of interacting with the α4β1 integrins was utilized to immobilize EVs on electrospun scaffolds (Hao et al., 2020). Similarly, tannic acid-mediated coupling of EVs on polyetheretherketone (PEEK)-based implants resulted in the controlled release of EVs, osteoimmunomodulation-mediated angiogenesis, and new bone formation (Fan et al., 2021). Alginate hydrogels have also been exploited to improve retention and achieve controlled release of dendritic cell (DC)-derived EVs, which induced the recruitment of regulatory T cells (Treg), shifted macrophages to reparative M2 phenotypes, and improved cardiac function (Zhang et al., 2021). Moreover, an array of biomaterials and scaffolds has been used to achieve the sustained and localized release of EVs for tissue repair (Liu et al., 2017a; Zhang et al., 2018a; Zhou et al., 2019; Brennan et al., 2020).

## Modulating the Injury Microenvironment to Enhance Stem Cell Retention and Engraftment

**FIGURE 6:**
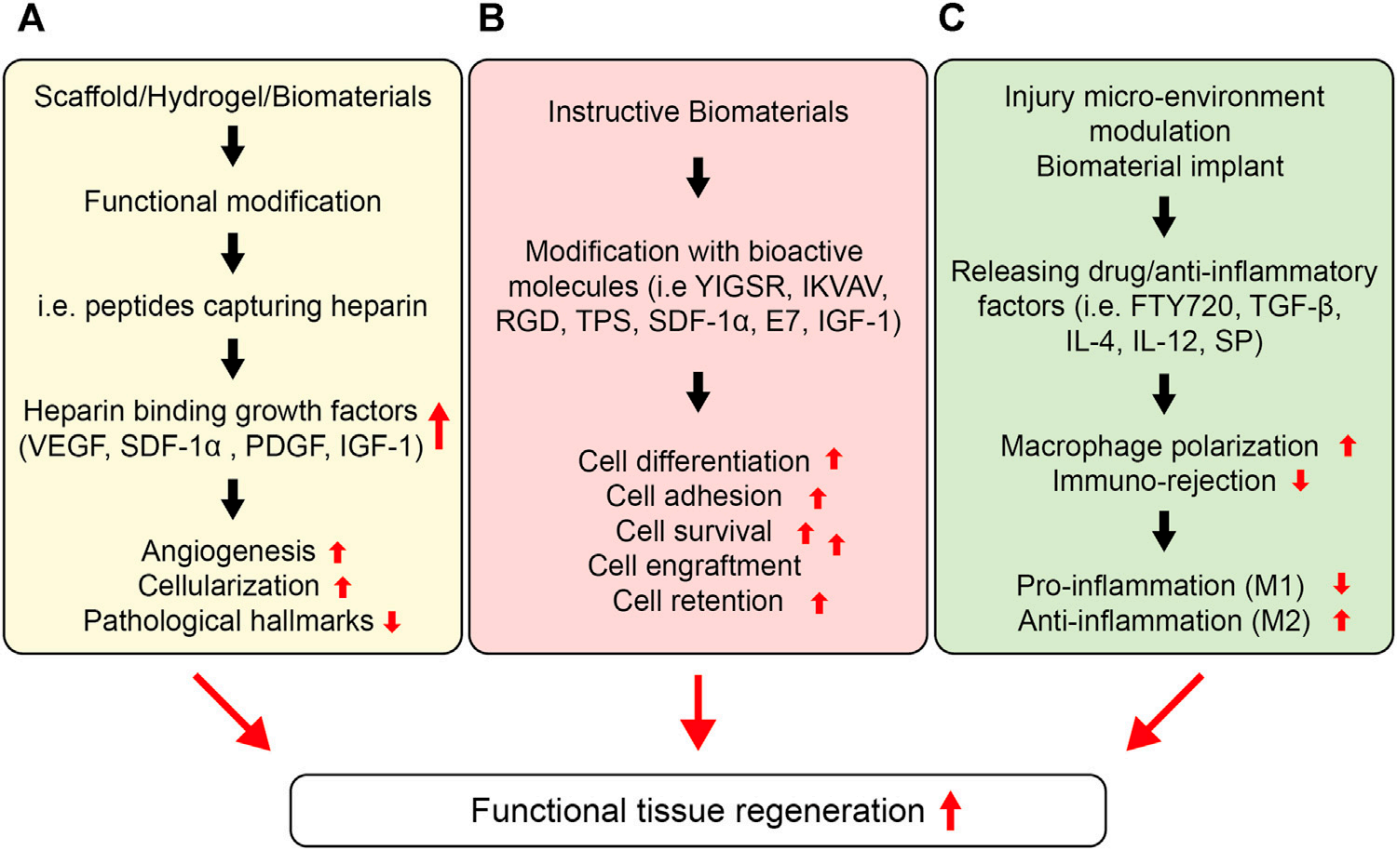
Strategies to enhance cell function for a functional tissue repair. **(A)** Stem cell function can be improved by sequestering proteins *in situ* via the functional design of biomaterials. Different types of peptides could be installed into biomaterials with the ability to endogenously capture cell-signaling proteins, such as heparin, which can then bind several heparin-binding growth factors, such as, VEGF, PDGF, IGF etc. **(B)** Instructive biomaterials designed by incorporating bioactive cues can also enhance cell survival, retention, and engraftment. Depending upon the nature of the signaling cue, they can enhance the stem cell differentiation, proliferation, or migration *in vitro* and *in vivo*, which can be central to the formation of *in vitro* tissue-engineered constructs as well as enhance cell engraftment *in vivo*. **(C)** The injury microenvironment can also be tuned to ameliorate stem cell function, including survival, retention, and engraftment. This can be achieved by releasing anti-inflammatory therapeutics and cytokines, including IL-4, IL-1, TGF-β1, rapamycin, resveratrol, SP etc. Consequently, these therapeutics and functional cues can lower the pro-inflammatory response and enhance the anti-inflammatory response overcoming the immune rejection, lowering the fibrosis, and enhancing the stem cell survival and retention as well as engraftment at the targeted site.

Because biomaterial-mediated inflammation of the injury microenvironment also leads to impaired tissue repair and implant failure, modulation of the injury microenvironment may have great potential as a way of improving the engraftment of transplanted cells and functional repair. A promising approach to modulating the injury microenvironment is to release inflammation-resolving biomolecules from the implants or sequester endogenous morphogens to ameliorate the tissue repair process (Figure 6 and Table 5). For instance, Gower et al. assembled TGF-β into layer-by-layer (LBL) scaffolds to resolve inflammation in the injury microenvironment and enhance the survival and retention of transplanted islets (Liu et al., 2016b). The localized release of TGF-β resulted in lower expression of inflammatory cytokines, such as TNF-α, IL-12, and MCP-1, thereby causing a significant decrease in leukocyte recruitment and higher survival of the transplanted islets as compared to those of the control group. Consequently, biomaterial-mediated modulation of the immune response has considerable potential to enhance cell function in cell-based therapies. Similarly, Cheng et al. leveraged microneedle patches to deliver cardiac stem cells (CSCs) to the heart (Tang et al., 2018). The microneedle patch was fabricated from polyvinyl alcohol (PVA), which provided a conducive environment for the survival of the transplanted CSCs and leveraged a pathway for the exchange of nutrients with the heart and the transportation of paracrine factors from the transplanted cells, thereby leading to better regeneration of the infarcted heart. Consequently, the cells transplanted using microneedle patches reduced scar tissue formation, increased blood vessel regeneration, and left ventricular wall stabilization in the infarcted heart. This technique could be further tailored to release paracrine factors in response to the injury microenvironment.

**TABLE 5:** Enhancing stem cell function by modulating the injury microenvironment.

Biomaterial	Cell/tissue type	Observed effect	References
TGF-β-loaded Layer-by-layer scaffolds	Islets	Cell survival ↑	Liu et al. (2016b)
		Inflammatory cytokines (e.g., TNF-α, IL-12, MCP-1) ↓	
		Leukocytes ↓	
Poly (vinyl alcohol) microneedle patch	CSCs	Paracrine factors secretion ↑	Tang et al., 2020
		Scar tissue ↓	
		Blood vessel regeneration ↑	
PEG-maleimide hydrogels	MSCs	ROS scavenging ↑	Martin et al. (2020)
		Cell survival ↑	
Immuno-protective device eluting alanine and glutamine	Islets	Cell survival ↑	Chendke et al. (2019)
		Immuno-protection of cells ↑	
Hydrogels encapsulating tacrolimus-eluting PLGA NPs	EPCs	Cell survival ↑	Li et al. (2018)
		Blood perfusion ↑	
Self-assembling peptides based hydrogels containing laminin-derived IKVAV motif	ESCs-derived cortical progenitors	Neuronal function ↑	Somaa et al. (2017)
		Motor function ↑	

TGF-β, transforming growth factor beta; PEG, poly(ethylene glycol); PLGA, poly(L-lactide-co-glycolide); NPs, nanoparticles; CSCs, cardiac stem cells; ESCs, embryonic stem cells; MSCs, mesenchymal stem cells; EPCs, endothelial progenitor cells; TNF-α, tumor necrosis factor alpha; MCP-1, monocyte chemoattractant protein 1; IL-12, interleukin 12.

To further tailor the injury microenvironment and improve the survival and engraftment of the transplanted cells, Duvall et al. designed reactive oxygen species (ROS)-scavenging hydrogels by installing dithiol polymers into PEG-maleimide-based hydrogels, which act as antioxidative biomaterials that quench ROS and enhance the survival of the transplanted cells (Martin et al., 2020). ROS-degradable poly (thioketal) (PTK) polymers were designed and leveraged as platforms for MSC transplantation. ROS-sensitive crosslinks enhanced the survival of MSCs by scavenging free radicals, and the transplanted cells showed significantly higher survival when this platform was used than when platforms containing only PEG hydrogels and enzymatically degradable hydrogels. These types of oxidation-sensitive biomaterials may afford novel hydrogels for modulating the injury microenvironment to benefit cell transplantation and drug delivery. Because hypoxia and nutrient deficiency cause cell loss upon transplantation and require strategies to improve the survival of the transplanted islets at the implantation site, Desai et al. fabricated an immunoprotective device capable of providing a sustained release of amino acids, namely, alanine and glutamine, which significantly improved the survival of the islets under nutrient-limiting conditions *in vitro* as well as in the subcutaneous space *in vivo* by conferring immune protection on the cells (Chendke et al., 2019). Amino acids were released from the films for up to 18 days. Grafts co-releasing alanine and glutamine increased cell survival *in vivo* by 33% compared to the control grafts, which lacked amino acids and showed 0% survival of the cells at 21 days post-transplantation. In another study, Wang et al. anchored immunosuppressant-releasing nanoparticles to hydrogels (Li et al., 2018). Tacrolimus was investigated as an immunosuppressant and bound to RADA16-modified PLGA nanoparticles (NPs). Tacrolimus was released for up to 28 days, which increased the survival of endothelial progenitor cells (EPCs) *in vitro* and *in vivo*. The NP-mediated release of tacrolimus lessened immune rejection; consequently, transplanted EPCs improved blood perfusion in a hind limb ischemia model. Shafiq et al. designed cardiac patches co-delivering neuropeptide substance P and insulin-like growth factor-1 domain C (IGF-1C) and evaluated their potential in an infarcted myocardium model in mice. IGF-1C was used to provide a cardio-protective environment to cardiomyocytes, which could reduce their apoptosis following myocardial infarction. The results showed that SP and IGF-1C acted synergistically to improve endogenous stem cell and progenitor cell recruitment and lowered the frequency of apoptosis *in vivo* (Shafiq et al., 2018) (Figure 5).

## Conclusion and Perspectives

Stem cell transplantation has received considerable attention from researchers. However, it is often accompanied by reduced survival, retention, and engraftment resulting from poor cell-cell and cell-ECM interactions during cell administration and post-transplantation. Consequently, to avoid cell loss during transplantation, many biomaterial platforms have been proposed, such as natural and synthetic polymer-based hydrogels, micro-and nano-spheres, and intelligent scaffolds. This work has substantially improved our understanding of the cell-cell and cell-matrix interactions as well as resulted in better cell survival *in vitro* and during transplantation (Figure 6). A wide variety of bioactive cues have been incorporated into biomaterials to modulate stem cell behavior *in vitro* and *in vivo*. Such bioactive cues not only help improve cell survival and growth during *in vitro* culture, but also stimulate cellular behavior post-transplantation. As cells perceive a gradient of morphogens and signaling cues *in vivo*, which may be tethered to the ECM or exist in soluble form, proper knowledge of these bioactive cues is necessary to understand cellular behavior. Several ECM-derived peptide sequences have been installed in biomaterials to affect cellular behavior *in vitro* and *in vivo*. Likewise, phage-display technology has garnered attention to screen peptides for modulating cellular fate. Screening biomaterials also holds great promise for providing cell-instructive platforms. The spatiotemporal presentation of signaling cues is also of fundamental importance, and has been studied by installing tissue microenvironment-responsive sequences into biomaterials to leverage timely cues to the transplanted cells and improve their survival, retention, and engraftment in the targeted tissues. Engineering biomaterials to leverage bioactive cues in a spatiotemporal fashion holds great promise and will require further attention from researchers.

The ECM helps instruct cell fate through tethered and soluble bioactive cues as well as mechanotransduction pathways. It has been determined that ECM stiffness regulates cell fate and drives tissue-specific cell lineage commitment. As the ECM exhibits non-linear mechanical behavior encompassing elastic and viscoelastic components, biomaterials that mimic the mechanical behavior of the ECM hold great promise for cell fate modulation and tissue engineering applications. Future research is therefore warranted to identify and develop such biomaterials, which may not only improve our understanding of cellular mechanotransduction but also leverage mechanotransduction pathways to discern development and pathological mechanisms. Interdisciplinary knowledge about the development of such biomaterials is needed to provide cell-instructive platforms.

The preconditioning and licensing of cells may help improve survival and engraftment after transplantation. As a library of growth factors that can drive tissue-specific cell lineage commitment has already been identified and that can be incorporated into biomaterials to improve cell survival and engraftment of the targeted tissues, a precise understanding of cell fate *in vivo* and cell engraftment into the targeted tissue is of pivotal significance. This has been realized by the integration of traceable magnetic nanoparticles and probes. As discussed above, cells perceive ECM-derived signaling cues in a continuum fashion by soluble and tethered ligands, and the judicious selection of biomaterials leveraging the on-off switch of signaling cues may help further investigate the cellular fate *in vitro* and *in vivo*. The identification of biomaterial platforms harboring and leveraging signaling cues in a cell-mediated fashion holds great promise. Lastly, because the fate of the transplanted cells is also governed by the injury or transplantation microenvironment and most of the transplanted cells are lost due to immune rejection, immune protection of the transplanted cells at the injury site may help improve cell survival and retention at the target site as well as their engraftment post-transplantation. This has been achieved by delivering immune-protective cues from biomaterial platforms in a spatiotemporal manner.
